# Palmitoylethanolamide and Related ALIAmides for Small Animal Health: State of the Art

**DOI:** 10.3390/biom12091186

**Published:** 2022-08-26

**Authors:** Giorgia della Rocca, Giovanni Re

**Affiliations:** 1 Department of Veterinary Medicine, Centro di Ricerca sul Dolore Animale (CeRiDA), University of Perugia, 06123 Perugia, Italy; 2 Department of Veterinary Sciences, Division of Pharmacology & Toxicology, University of Turin, 10095 Grugliasco, Torino, Italy

**Keywords:** ALIAmides, dogs, cats, atopic dermatitis, osteoarthritis, mast cells, palmitoylethanolamide, Adelmidrol, palmitoylglucosamine

## Abstract

ALIAmides are a family of fatty acid amides whose name comes from their mechanism of action, i.e., the Autacoid Local Injury Antagonism (ALIA). Actually, the ALIAmide parent molecule, palmitoylethanolamide (PEA), is locally produced on demand from a cell membrane precursor in order to control immune-inflammatory cell responses, avert chronic non-resolving inflammation, and limit the resulting clinical signs. ALIAmide sister compounds, such as Adelmidrol and palmitoylglucosamine, share mechanisms of action with PEA and may also increase endogenous levels of PEA. Provided that their respective bioavailability is properly addressed (e.g., through decreasing the particle size through micronization), exogenously administered ALIAmides thus mimic or sustain the prohomeostatic functions of endogenous PEA. The aim of the present paper is to review the main findings on the use of ALIAmides in small animals as a tribute to the man of vision who first believed in this “according-to-nature” approach, namely Francesco della Valle. After briefly presenting some key issues on the molecular targets, metabolism, and pharmacokinetics of PEA and related ALIAmides, here we will focus on the preclinical and clinical studies performed in dogs and cats. Although more data are still needed, ALIAmides may represent a novel and promising approach to small animal health.

## 1. Introduction

ALIAmides are a family of fatty acid amides sharing a common mechanism of action, i.e., the autacoid local injury antagonism (ALIA), originally proposed in the mid-1990s by the late Nobel prize winner Rita Levi Montalcini [1]. The term “autacoid” comes from the Greek “autos” (self) and “acos” (healing or remedy) and refers to cell-produced factors that act locally near their site of synthesis [2]. In particular, the autacoid mechanism of ALIAmides serves auto-protective purposes through the down-modulation of cell hyperactivity (mainly immune cells), thus controlling inflammatory responses and limiting tissue damage [3]. It was originally observed that the ALIAmide parent molecule, palmitoylethanolamide (PEA), down-modulates rat mast cell behavior after challenge [1,4], as later confirmed in companion animals [5,6,7]. Different cell populations were also shown to be targets of PEA, with macrophages, keratinocytes, T and B cells, and glial cells being negatively controlled by PEA once overactivated [8,9,10,11,12,13,14,15,16,17,18].

Palmitoylethanolamide is a body’s own (endogenous) N-acylethanolamine, produced “on demand” by several cell types, including mast cells, astrocytes, and microglia [19,20,21]. Interestingly, the autoprotective function of PEA was first suggested in dogs. It was indeed found that (i) the canine myocardium produces PEA in response to ischemic injury [22,23], and (ii) the canine brain possesses the biosynthetic and degradative machinery for PEA [24]. Since the 1980s, knowledge has advanced considerably in the field of ALIAmides, mainly due to the renewed interest in these molecules driven by the discovery of the PEA congener and the endocannabinoid mediator anandamide arachidonoylethanolamide (AEA) [25].

**Figure 1:**
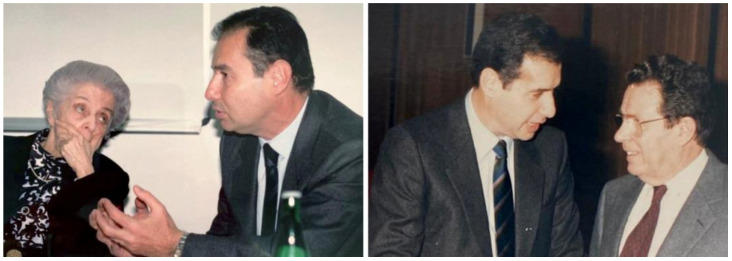
Francesco della Valle in the 1990s during brainstorming with his main scientific mentors, namely Rita Levi Montalcini (**left**) and Erminio Costa (**right**).

In those days, an enlightened man, Francesco della Valle (to whom the present special issue is dedicated), was launching his own science-driven entrepreneurial activity in the field of human and animal health, focused on innovation and networking [26]. During his previous experience in managing a pharmaceutical firm, he had been actively cooperating with two eminent scientists, Rita Levi Montalcini [27] and Erminio Costa [28,29] (Figure 1).

Both of them repeatedly invited della Valle to orientate the focus and efforts toward biological modulation mechanisms while learning from nature how to design a strategy of modulation [30,31]. Accordingly, della Valle based his strategic business plan on a “hypothetical-deductive” approach to inflammation and pain, according to regulatory pathways laid down by nature and intended to maintain a homeostatic balance in the body when challenged by stress or injury. This was the ALIAmide project. Although the historical view of ALIAmides is beyond the scope of the present review, it must be acknowledged that the ALIAmide story began in this particular framework, and most of the research data that will be reviewed here were born within it.

**Figure 2:**
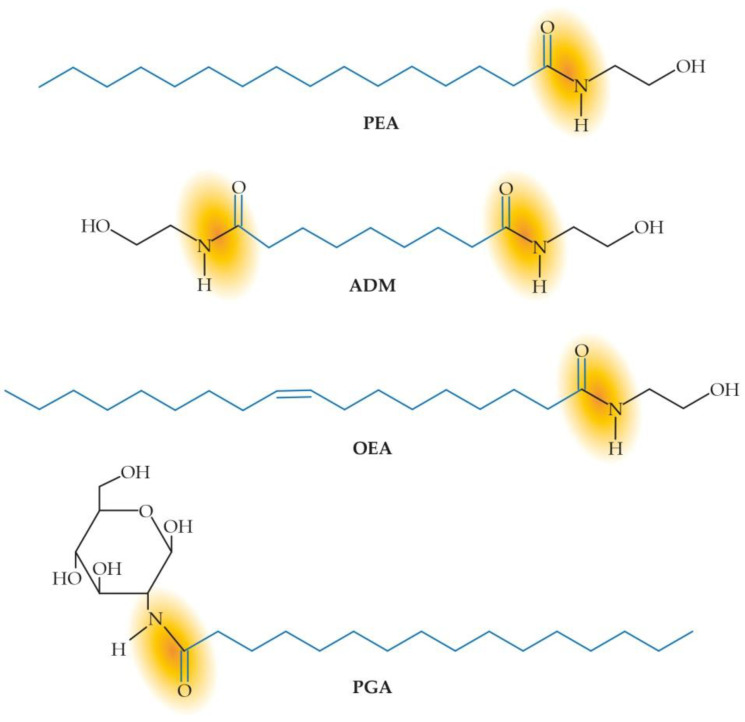
Chemical structure of the main ALIAmides. The amide bond (yellow shadow) and the fatty acid (blue color) are highlighted. ADM = Adelmidrol, OEA = oleoylethanolamide, PEA = palmitoylethanolamide, PGA = palmitoylglucosamine.

Besides PEA, ALIAmides currently comprise several lipid compounds, ranging from Adelmidrol (the diethanolamide derivative of azelaic acid) to palmitoylglucosamine (PGA), oleoylethanolamide, and many others (Figure 2).

Their respective mechanisms of action have been (and still are being) investigated and appear to be profoundly interconnected to the parent compound PEA, which is by far the most studied ALIAmide [3,32,33]. A brief overview of their molecular mechanisms will be given in the following paragraphs.

A large body of evidence has been accumulating on the prohomeostatic functions of ALIAmides in several diseases sustained by non-resolving inflammatory and neuroinflammatory responses. The findings have been reviewed by several excellent papers, to which the reader is encouraged to refer [3,32,33,34,35,36,37]. After addressing a few general key points on ALIAmides, here we will focus exclusively on the main studies performed on small animals.

## 2. Mimicking and Supporting the Healing Power of Nature

Palmitoylethanolamide is produced starting from a glycerophospholipid precursor in the cell membrane and degraded by two amidases located in the cell membrane and lysosome, respectively, i.e., the fatty acid amide amidase (FAAH) and N-acylethanolamine acid amidase (NAAA) [38,39,40,41]. Although the endogenous levels of PEA are strictly regulated by these biosynthetic and degradative metabolic pathways [38], great deals of evidence suggest that PEA metabolism may be disturbed under certain conditions, such as chronic inflammatory disorders [42]. Indeed, the local levels of PEA change during disease states, and decreased levels are considered to contribute to disease development [8,43,44]. For example, a significant decrease in the local level of PEA has been found in different chronic pain models [45,46,47] as well as in human patients affected by visceral and somatic pain [48,49,50]. Interestingly, it was also shown that normalizing PEA levels through the inhibition of PEA degradative pathways resulted in reduced inflammation and pain relief in a rat model of osteoarthritis pain [47].

On the other side, PEA levels may increase in response to cell damage, as shown in epidermal cells subjected to UV irradiation [51] and the lesional skin of privately-owned dogs affected with atopic dermatitis [52] as well as the colons of dogs with chronic enteropathy [53].

It is currently accepted that changes in PEA levels are either suggestive of a loss of protection against inflammation/pain (i.e., decreased levels) or a compensatory synthesis in the attempt to limit disease severity (i.e., increased levels). Accordingly, the exogenous administration of PEA to effectively ‘top up’ the body’s own supply is regarded as a promising approach [54]. Interestingly, other ALIAmides, such as Adelmidrol and PGA, have recently been found to increase the endogenous levels of PEA [55,56,57].

## 3. A Brief Insight into PEA Metabolism and Molecular Targets

As mentioned above, the biosynthesis of PEA occurs “on demand” in the cell membrane through the enzymatic hydrolysis of its glycerophospholipid precursor N-acyl-phosphatidylethanolamine [39,40]. Although early studies suggested the existence of a facilitated membrane transport [19,58], PEA can flip between the inner and outer leaflets of the plasma membrane thanks to its lipophilic nature [59]. Indeed intracellular binding proteins (i.e., fatty acid binding proteins and heat-shock proteins) are required for PEA trafficking within the cytosol [60]. Binding proteins transport PEA to catabolic enzymes (e.g., FAAH and NAAA) [41] and effector proteins [61,62,63].

Among the latter, the nuclear peroxisome proliferator-activated receptor alpha (PPARα) is of particular interest because it negatively interferes with inflammatory gene expression by regulating the IκBα/NF-κB pathway [64]. PPARα is not the only molecular target responsible for the prohomeostatic properties of PEA [65,66,67,68,69], as many other receptors are being increasingly recognized as mediating PEA functions, such as the GPR55 (G-protein-coupled receptor 55) [70,71], cannabinoid receptors type 1 and 2 (CB1 and CB2) [33,57,72,73] as well as the so-called “pain receptor” [74], i.e., the transient receptor potential vanilloid 1 (TRPV1) [75,76,77,78].

Interestingly, this heterogeneous family of PEA molecular targets is being extensively studied in companion animals, with their distribution being confirmed in several canine and feline cell types [79,80,81,82,83,84,85,86,87,88,89,90,91,92], as recently reviewed [3,32,93].

**Figure 3:**
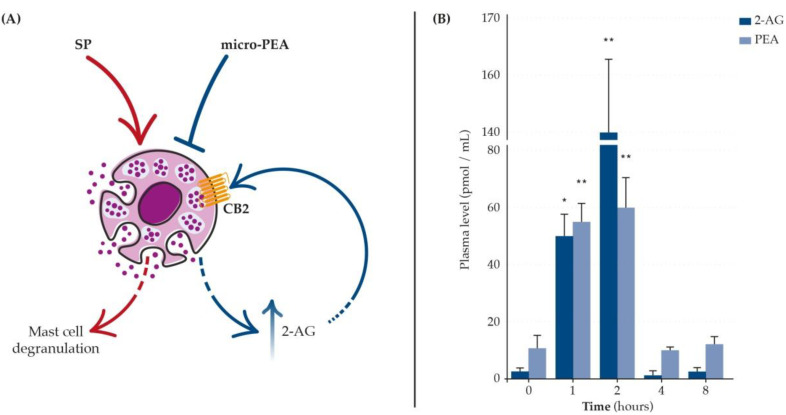
PEA may act on its molecular targets either directly or indirectly by increasing the agonism toward endocannabinoid receptors for which it has a low affinity. The latter mechanism is referred to as the “entourage effect”. The figure illustrates the in vitro (**A**) and in vivo (**B**) demonstrations of the entourage effect of bioavailable formulations of PEA (i.e., micro-PEA, please see next paragraph for further details) through increasing the levels of the endocannabinoid 2-AG. (**A**) Indirect agonism of micro-PEA on CB2 underlies the inhibitory effects on SP-induced mast cell degranulation, mediated by the stimulation of 2-AG biosynthesis [57]. (**B**) Following a single dietary supplementation with micro-PEA to hypersensitive Beagle dogs, not only plasma levels of PEA but also plasma levels of 2-AG significantly increase (* *p* < 0.05 and ** *p* < 0.001 versus the basal levels, time 0) [3]. (**B**) is slightly modified from [3]. 2-AG = 2-arachidonoylglycerol, CB2 = cannabinoid receptor type 2, micro-PEA = micronized or ultramicronized palmitoylethanolamide, SP = substance P.

Notably, while PEA is a direct agonist of PPARα [66], its action on CB1, CB2, and even TRPV1 is indirect [73,76,77,78]. In particular, PEA can activate these latter three receptors thanks to its ability to (i) elevate their levels, (ii) reduce their degradation, or (iii) increase the receptor affinity of endocannabinoids, like AEA and 2-arachidonoylglycerol (2-AG) [35,57,72,73,76,78]. The mechanism has been termed the “entourage effect” [73,76,78] (Figure 3) and has been specifically shown in dogs [72]. In Beagle dogs, orally administered bioavailable micro-PEA (i.e., ultra-micronized, see below) resulted in a significant and up to ~20-fold increase in the plasma levels of 2-AG [72] (Figure 3B).

To date, the molecular mechanisms of other ALIAmides are much less investigated than PEA’s. Besides increasing PEA levels, as previously mentioned, these fatty acid amides are suggested to interact with different receptors. PGA, for example, is considered to exert its protective function through a toll-like receptor 4 antagonism [94], while the precise molecular targets of Adelmidrol are still debated [55,95].

## 4. Key Pharmacokinetic Issues

A key aspect that has to be taken into account when dealing with the use of ALIAmides for health purposes is their respective physicochemical features. Some ALIAmides are more appropriate for oral use, while others are particularly suitable for topical applications thanks to their amphipathic nature (e.g., Adelmidrol) [95,96].

PEA and PGA are both highly lipophilic compounds (log *p* > 5) [97,98], with their oral use being limited by their intrinsic low dissolution rate, absorption, and bioavailability [98,99]. Particle size reduction is one of the most compelling and practical strategies for improving pharmacokinetics and boosting functional properties following oral administration [100,101]. Provided the route of administration is oral, most of the studies presented below investigated “micro-PEA” and “micro-PGA” accordingly. Micro-ALIAmides result from micro-grinding a particular ALIAmide—either alone or together with adjuvants (typically antioxidants)—in order to downsize the particles to diameters in the range of 0.6–10 μm. Indeed, after the administration of micro-PEA, the plasma concentration of PEA was significantly higher compared to unprocessed (naïve) PEA [98]. Accordingly, superior effects have been shown for micro-PEA and micro -PGA compared to naïve PEA and PGA, respectively, in different inflammatory disease models [97,102,103].

Specifically, in dogs, a single oral administration of micro-PEA resulted in a five-fold increase in PEA plasma levels, with a peak between 1 and 2 h [72,104]. Interestingly, plasma levels correlated well with the clinical effects at different timepoints, although the latter lasted longer than the plasma elevation of PEA [104]. This was considered to depend on the ability of PEA to up-regulate the levels or enhance the action of other related bioactive endocannabinoids [104], according to the so-called “entourage hypothesis” briefly outlined in Figure 3.

## 5. Preclinical and Clinical Results in Small Animals

### 5.1. Dermatological Field

So far, most of the veterinary research on ALIAmides has been focused on the dermatological field [105]. Ex vivo and in vitro studies, performed on feline and canine skin mast cells, respectively, have confirmed that micro-PEA down-modulates allergic hyperactivity, prominently decreasing mediator release (i.e., degranulation) [5,7]. The ability of micro-PEA to down-modulate mast cell degranulation was also recently shown in canine skin organ cultures challenged with different concentrations of compound 48/80 (a well-known secretagogue which triggers mast cell degranulation) [6]. Not only did micro-PEA significantly counteract the increase of degranulating mast cells, but it also lowered the histamine content within the culture medium and the diameter of epidermal blood capillaries [6].

Moreover, down-modulation of skin mast cell releasability was observed in canine skin wounds (punch biopsies) topically treated with the ALIAmide Adelmidrol (2%) [106], with a parallel improvement in wound healing being detected [107].

Moving to in vivo studies, a growing body of evidence confirms that ALIAmides can efficiently benefit veterinary patients with hypersensitive skin disorders. In a double-blinded placebo-controlled cross-over study performed on dogs with experimental allergic dermatitis, the dietetic supplementation with micro-PEA at 15 mg/kg/day for 7 days delayed the development of clinical signs (i.e., pruritus and skin lesions) compared to the placebo-treated group [108]. Moreover, in a canine model of skin allergy, a single oral administration of micro-PEA (3, 10, and 30 mg/kg) significantly reduced the antigen-induced wheal area, with a maximum inhibitory effect at a 10 mg/kg dose [104]. Interestingly, topical application of Adelmidrol (2%) for 3 and 6 consecutive days gave similar results in terms of allergic wheal inhibition [96].

On the clinical side, two studies were performed on allergic cats. The first one investigated feline patients with eosinophilic plaques and eosinophilic granuloma, orally given micro-PEA (10 mg/kg daily) for 1 month as the sole intervention. Clinical improvement of pruritus, erythema, alopecia, and eosinophilic lesions was observed in 67% of them, with no side effects or adverse reactions being reported [7]. The second was conducted in 60 allergic cats with the aim of evaluating whether micro-PEA (15 mg/kg) could delay the relapse of clinical signs after steroid withdrawal [109]. A significant difference in the mean time-to-flare between the treated and placebo group was observed (40.5 days in the micro-PEA group vs. 22.2 days in the placebo group), suggesting that the ALIAmide exerts an excellent proactive function in preventing feline allergic flares after steroid withdrawal [109].

Some interesting clinical trials were also performed on allergic dogs. A double-blinded randomized placebo-controlled cross-over study in privately-owned dogs with either food-induced or non-food-induced atopic dermatitis showed that dietary integration with micro-PEA (15 mg/kg daily for 45 days) significantly decreased the severity of clinical signs (as assessed by the Canine Atopic Dermatitis Extension and Severity Index) [110].

An open multicentric study performed in 160 client-owned dogs with non-seasonal atopic dermatitis orally administered micro-PEA (10 mg/kg daily for 56 days) confirmed the ability of the ALIAmide to benefit allergic patients [111]. Pruritus (as measured on a Visual Analogue Scale) and clinically assessed skin lesions (Canine Atopic Dermatitis Lesion Index) were significantly reduced by the study end. Moreover, 45% of dogs reached the quality of life values described for healthy animals [111].

Finally, an open-label observational study was performed in privately-owned dogs with atopic dermatitis and pruritus lasting longer than 4 weeks, topically treated with Adelmidrol (2%) twice daily for 30 days. Not only a significant decrease in pruritus and erythema (both on owner and veterinarian assessment) was observed, but body odor and quality of life markedly improved by the study’s end [112].

### 5.2. Other Health Needs

Although studies in small animals are still scarce, there is growing evidence that endocannabinoid-like ALIAmides play key roles in the health of different body organs, such as the gastrointestinal tract [113,114] and the nervous system [32,34,37], as well as the upper and lower urinary tract [115,116,117,118] and the musculoskeletal system [97,119,120]. In addition, the deep involvement of ALIAmides in obesity-induced metainflammation is becoming increasingly evident [69,113,121,122,123].

Actually, a preliminary study in dogs affected with chronic diarrhea demonstrated that dietetic supplementation with micro-PEA (10 mg/kg for 30 days) reduced the Canine Inflammatory Bowel Disease Activity Index (CIBDAI) score [53], in line with recent findings from animals with experimentally-induced colitis [124]. According to the experimental studies, the enteroprotective effect of PEA may depend upon the direct and indirect activation of PPAR-α and CB2 receptors [124,125,126,127,128,129,130], whose expression has been recently confirmed in the canine and feline gastrointestinal tract [86,87].

Interestingly, a dietetic supplement containing micro-PEA was also described to benefit a Syrian hamster with urolithiasis and diminish the disease recurrence after surgical treatment [131]. Moreover, micro-PGA has recently been shown to decrease inflammation and pain in a murine model of feline interstitial cystitis [132].

In the musculoskeletal field, an open-field trial on client-owned adult dogs with chronic osteoarthritis and persistent lameness has recently been performed. Dogs were supplemented for 4 weeks with a complementary feed containing PEA co-ultramicronized with the natural antioxidant quercetin (i.e., PEA-q, 24 mg/kg body weight). The severity of chronic pain and its interference with the dog’s normal functioning significantly decreased as assessed with the Canine Brief Pain Inventory (CBPI) questionnaire. Moreover, lameness (either assessed on a 0–4 clinical scale or through a dynamic gait analysis) significantly improved [133].

Dogs with osteoarthritis also benefited from a long-term dietary integration with the ALIAmide PGA co-micronized with curcumin, administered as an add-on to conservative measures. One trial has been performed [134], where micro-PGA was added for 2 months to the individual management plan of 181 dogs with osteoarthritis. A significant decrease in lameness and pain as assessed by the veterinarian was observed. Moreover, owner-evaluated mobility impairment and pain behaviors also improved [134].

It is finally noteworthy that the topical administration of an Adelmidrol (2%) mucoadhesive gel in combination with dental prophylaxis resulted in less gingival inflammation and longer duration of dental scaling benefits in treated dogs compared to match untreated group [135].

**Figure 4:**
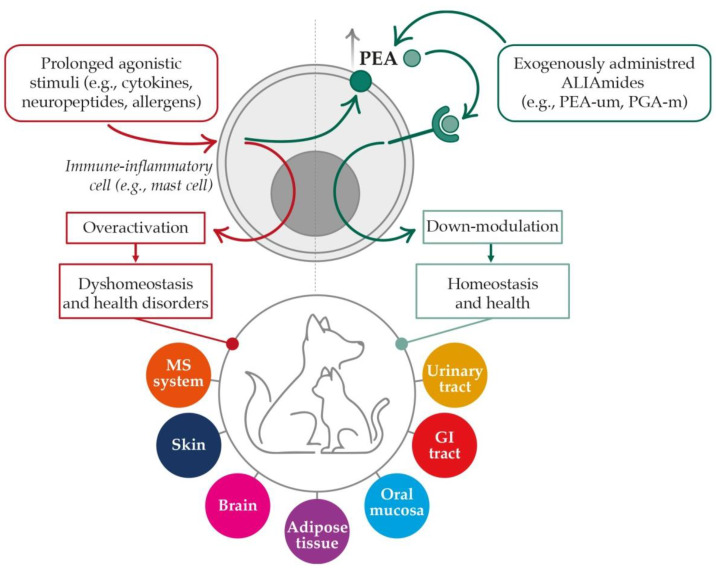
ALIAmides for small animal health—a global view. Upon prolonged stimulation, immune-inflammatory cells may become overactivated. If uncontrolled, their beneficial protective responses may instead turn harmful, leading to local dyshomeostasis and health disorders. In order to control the risk, autoprotective mechanisms are activated. The local production of PEA starting from a glycerophospholipid precursor (dark green circle) represents one of them. Once produced, PEA (light green circle) serves as a signaling molecule through its direct and indirect interactions with multiple receptor targets resulting in cell down-modulation. Local homeostasis and body health are maintained accordingly. Exogenously administered ALIAmides mimic or sustain the autoprotective mechanism described above, mainly through restoring endogenous PEA levels. The main organs and body tissues purportedly benefiting from the aforementioned mechanism are listed in the colored circles on the bottom. GI = gastrointestinal, MS = musculoskeletal, PEA-um = ultramicronized palmitoylethanolamide, PGA-m = micronized palmitoylglucosamine.

Taken together, the data from preclinical and clinical trials point towards the promising role of ALIAmides in small animal health (Figure 4). Moreover, the presence of PEA and OEA, as well as other ALIAmides in food sources [136], in addition to their robust safety profile [36,97,137], are the foundation for their dietary use. Accordingly, several complementary feeds for dogs and cats have been developed and are being marketed in Europe and North America.

## 6. Conclusions

Although the field is still in its infancy, the studies presented in this review highlight the promise that ALIAmides might play a broad role in small animal health. Their physiological prohomeostatic functions represent a key rationale for their use in promoting animals’ health through an “according-to-nature” approach, i.e., mimicking or supporting the physiological mechanisms to maintain homeostasis.

Although further clinical studies are needed, ALIAmide-based products—either used as a sole intervention or associated with standard drugs—are emerging as a new and promising approach to veterinary patients.

## Data Availability

Not applicable.
